# Loss of function of the mitochondrial peptidase PITRM1 induces proteotoxic stress and Alzheimer’s disease-like pathology in human cerebral organoids

**DOI:** 10.1038/s41380-020-0807-4

**Published:** 2020-07-07

**Authors:** María José Pérez, Dina Ivanyuk, Vasiliki Panagiotakopoulou, Gabriele Di Napoli, Stefanie Kalb, Dario Brunetti, Rawaa Al-Shaana, Stephan A. Kaeser, Sabine Anne-Kristin Fraschka, Mathias Jucker, Massimo Zeviani, Carlo Viscomi, Michela Deleidi

**Affiliations:** 1 German Center for Neurodegenerative Diseases (DZNE), Tübingen, Germany; 2 Department of Neurodegenerative Diseases, Hertie Institute for Clinical Brain Research, University of Tübingen, Tübingen, Germany; 3 Department of Medical Biotechnology and Translational Medicine, University of Milan, Milan, Italy; 4 Department of Cellular Neurology, Hertie Institute for Clinical Brain Research, University of Tübingen, Tübingen, Germany; 5 DFG NGS Competence Center Tübingen, 72076 Tübingen, Germany; 6 Institute of Medical Genetics and Applied Genomics, University of Tübingen, 72076 Tübingen, Germany; 7 MRC-Mitochondrial Biology Unit, Cambridge CB2 0XY, UK

## Abstract

Mutations in pitrilysin metallopeptidase 1 (PITRM1), a mitochondrial protease involved in mitochondrial precursor processing and degradation, result in a slow-progressing syndrome characterized by cerebellar ataxia, psychotic episodes, and obsessive behavior, as well as cognitive decline. To investigate the pathogenetic mechanisms of mitochondrial presequence processing, we employed cortical neurons and cerebral organoids generated from PITRM1-knockout human induced pluripotent stem cells (iPSCs). PITRM1 deficiency strongly induced mitochondrial unfolded protein response (UPR^mt^) and enhanced mitochondrial clearance in iPSC-derived neurons. Furthermore, we observed increased levels of amyloid precursor protein and amyloid β in PITRM1-knockout neurons. However, neither cell death nor protein aggregates were observed in 2D iPSC-derived cortical neuronal cultures. On the other hand, over time, cerebral organoids generated from PITRM1-knockout iPSCs spontaneously developed pathological features of Alzheimer’s disease (AD), including the accumulation of protein aggregates, tau pathology, and neuronal cell death. Single-cell RNA sequencing revealed a perturbation of mitochondrial function in all cell types in PITRM1-knockout cerebral organoids, whereas immune transcriptional signatures were substantially dysregulated in astrocytes. Importantly, we provide evidence of a protective role of UPR^mt^ and mitochondrial clearance against impaired mitochondrial presequence processing and proteotoxic stress. Here, we propose a novel concept of PITRM1-linked neurological syndrome whereby defects of mitochondrial presequence processing induce an early activation of UPR^mt^ that, in turn, modulates cytosolic quality control pathways. Thus, our work supports a mechanistic link between mitochondrial function and common neurodegenerative proteinopathies.

## Introduction

Mitochondrial dysfunction has been described as a common hallmark of neurological diseases [1]. However, in these conditions, mitochondria are often considered a secondary target rather than the actual disease driver. We have recently described three independent families carrying missense loss of function mutations in pitrilysin metallopeptidase 1 (PITRM1), resulting in an age-dependent, progressive, neurological syndrome [2, 3]. Patients suffer from progressive cerebellar dysfunction leading to cerebellar atrophy, with psychiatric manifestations including obsessive behavior, anger attacks, and psychosis [2, 3]. Interestingly, some of these patients showed a deterioration of their cognitive functions with a slow progression until their late sixties [2, 3]. Human PITRM1, also known as presequence peptidase (hPreP), is a nuclear-encoded mitochondrial gene expressed in a number of tissues, including muscles and different brain regions, e.g., the cortex, hippocampus, cerebellum, and tectum [4]. PITRM1 was initially identified in *Arabidopsis thaliana* as a protease that degrades targeting peptides in mitochondria and chloroplasts [5]. Human PITRM1 is a mitochondrial matrix enzyme that digests the mitochondrial-targeting sequences (MTS) of proteins imported across the inner mitochondrial membrane after their cleavage from protein precursors by the mitochondrial matrix presequence peptidase (MPP). When the MTS are not properly degraded, they accumulate within the mitochondrial matrix, causing dissipation of the mitochondrial membrane potential and mitochondrial dysfunction [6–8]. The incomplete processing of mitochondrial preproteins leads to their destabilization, resulting in alterations of mitochondrial proteostasis [9]. In vitro studies using recombinant PITRM1 have shown that, besides MTS, the enzyme is also involved in the degradation of short, unstructured peptides, and amyloid beta (Aβ) peptides [9–12]. Interestingly, Aβ peptides inhibit the activity of CYM1, the PITRM1 orthologue in yeast, leading to the accumulation of precursor proteins [9]. Experimental work in mouse models supports a causal role of PITRM1 in neurodegenerative dementia, whereby the loss of PITRM1 function leads to a progressive, neurodegenerative phenotype characterized by hindlimb clasping, impairment in motor coordination, and basal-ganglia-related movement control [2]. Interestingly, PITRM1-deficient mice exhibit an age-dependent accumulation of amyloid precursor protein (APP) and Aβ deposits within the brain [2], suggesting a link between defects of mitochondrial proteostasis and adult-onset neurodegenerative dementia. However, due to the embryonic lethality observed in complete PITRM1--knockout mice, the exact role of PITRM1 in brain homeostasis and disease could not be studied [2]. Because PITRM1 is involved in the degradation of MTS, as well as Aβ peptides [9], the pathomechanisms of neurodegeneration linked to the loss of PITRM1 function could, in principle, be due to either an accumulation of Aβ peptides in mitochondria, MTS-driven toxicity, or a combination of both. Identifying the mechanisms that lead to neurodegeneration in primary mitochondrial diseases characterized by defects of mitochondrial proteostasis, such as PITRM1-linked neurological syndrome, may help clarify the long-debated but still unresolved involvement of altered mitochondrial function in neurodegenerative dementia. To examine PITRM1-related pathogenetic mechanisms, we generated PITRM1-knockout human induced pluripotent stem cells (iPSCs) and examined the role of mitochondrial function and proteostasis using 2D neuronal and 3D brain-organoid model systems.

## Methods

### Generation of PITRM1-knockout human iPSCs

The control iPSCs (from a female non-affected control donor, 80 years) used in this study were previously generated and characterized [13]. All cells used in the study were derived from patients who had signed an informed consent approved by the Ethics Committee of the Medical Faculty and the University Hospital Tübingen. iPSCs were kept in culture in hESC medium [13]. SgRNAs targeting exon 3 and exon 4 of the PITRM1 gene were designed using CRISPR Guide Design Tools (former www.crispr.mit.edu) and purchased from Metabion International AG (exon 3 top AGGAGCCAGGTATTTACACC, exon 3 bottom GGTGT AAATACCTGGCTCCT, exon 4 top TTGAGCATACCGT CCTTTGT, exon 4 bottom ACAAAGGACGGTATGCT CAA). SgRNAs were cloned into the pSpCas9(BB)-2A-Puro plasmid containing the sgRNA scaffold and puromycine-resistance under the U6 promoter (Addgene plasmid #48139). Colonies that successfully integrated sgRNA into the backbone plasmid were screened and confirmed by Sanger sequencing using the U6 promoter region primer. SgRNA/Cas9 plasmid was delivered into cells using the Nucleofector Amaxa system (Lonza Biosciences, NC, USA). In brief, iPS cells were dissociated with Accutase (Sigma-Aldrich, MO, USA) and 10^6^ iPSCs were nucleofected with 6 μg of each sgRNA plasmid. The cells were then replated on MEF cells in hESC medium, without P/S, supplemented with 10 μM ROCK Inhibitor Y-27632 2 HCl. After recovery, cells were replated at a density of 500 cells/cm^2^ for single-cell subcloning. After recovery, iPSCs were clonally expanded and the genomic deletion was assessed by PCR and Sanger sequencing (exon 3 Fw TTCAGGCAGAAAAGCCAGTT, exon 4 Rv ACTGAATT CCAGTGGGTGTGC). The analysis of possible off-target effects was performed using CRISPR Design Tools. Sequencing primers for off-target effects: (5′–3′): PPIL2 NM_148175 Fw CCTCATGCCCTGCTTGACTC, Rv CAGGGAGCACTGTCCCAATTT; NR1D1 NM_021724 Fw CAAACGAGCACACACCACAG, Rv GCTGCCCCC TTGTACAGAAT; KDEL2 NM_001100603 Fw TTGGT GGTGGTTATGCCTCA, Rv ACCACCAGAAACTCCAC TCG; FAM120A NM_014612Fw TCCTGCGGTTCTT GTCCTCTA, Rv GCATGAATGTGTCTTCTCTGGC; TT LL2 NM_031949 Fw GTGGGAGGCTGTGTGGTATT, Rv TCAAGTCCCTACCTGTGCCA; SUCO NM_014283 Fw AATCTGGTACTATTCCGATAGCCAA, Rv CCATT CAAACAGGACACTGCTG.

### Cortical neuronal differentiation

For the induction of cortical neurons, we used an embryoid body (EB)-based differentiation protocol, with minor modifications [14]. iPSC colonies were manually chosen and grown for 4 d in EB media (20% KO serum replacement in DMEM/F12 medium, 1% NEAA, 1% P/S). On day 5, EBs were plated onto a Matrigel-coated (Corning, NY, USA) plate and grown for 4 more days in N2B27 media (DMEM/F12, 1× N2, 1× B27-RA, 1% NEAA, 1% P/S, 20 ng/mL bFGF). For the first 8 differentiation days, the cells were grown in the presence of 10 μM SB431542 (Ascent Scientific, UK) and 2.5 μM dorsomorphin (Sigma-Aldrich, MO, USA). After 8 days, neural rosettes were lifted with dispase, replated onto a matrigel-coated plate, and grown in N2B27 media. Secondary or tertiary rosettes were manually dissected to purify neural progenitor cells (NPCs). For cortical differentiation, NPCs were dissociated with Accutase and seeded at a density of 1000 cells per mm^2^ on matrigel-coated plates in a neuronal differentiation medium consisting of DMEM/F12,1× Glutamax, 1× N2, 1× B27-RA, 20 ng/mL BDNF (Peprotech, NJ, USA), 20 ng/mL GDNF, 1 mM dibutyryl-cyclic AMP (Sigma-Aldrich, MO, USA), and 200 nM ascorbic acid (Sigma-Aldrich, MO, USA). The medium was replaced every other day.

### Cerebral organoids culture and immunohistochemistry

Cerebral organoids were generated and maintained using the protocol described by Lancaster et al. [15]. Where indicated, cerebral organoids were treated with 500 nM ISRIB or 500 μM nicotinamide mononucleotide (NMN) (both from Sigma-Aldrich, MO, USA) daily, from DIV 20 to DIV 50 or from DIV 45 to DIV 50, respectively. For immunostaining, organoids were washed with PBS, fixed in 4% PFA for 15 min and then equilibrated in 30% sucrose in PBS overnight at 4 °C. The next day, the organoids were embedded in blocks with a mixture of 10% sucrose/7.5% gelatin, snap-frozen, and kept at –80 °C until cryosections were prepared using a Leica CM 1900 instrument (Leica, Germany) with a 20-μm thickness. Sections were then permeabilized and blocked with 10% normal goat serum in PBS in 0.5% Triton X-100. Primary antibody incubations were performed at 4 °C overnight, followed by three 10-min washes in PBS and staining with Alexa Fluor secondary antibodies (1:1000, Invitrogen, MA, USA) at the room temperature for 1 h. Primary antibodies included rat anti-CTIP2 (1:500, Abcam #ab18465, UK), rabbit anti-TBR1 (1:500, Abcam ab31940, UK), mouse anti-β-III-tubulin (1:1000, BioLegend, CA, USA. Previously Covance #PRB-435P), chicken anti-MAP2 (1:3000, BioLegend #PCK-554P, CA, USA), mouse anti phospho-tau AT8 (Ser202, Thr205) (1:1000, Invitrogen #MN1020, MA, USA), mouse anti-APP (1:500, Santa Cruz Biotechnology #sc53822, TX, USA), mouse anti-ubiquitin (1:100, Merck #MAB1510, NJ, USA), rabbit anti-cleaved caspase-3 (1:500, Cell Signaling Technology #9664T, MA, USA), and Phalloidin-iFluor 594 Reagent (1:1000, Abcam #ab176757, UK). Sections were stained with 10 μM thioflavin-T (Sigma-Aldrich #T3516, MO, USA) or 10 μM BTA-1 (Sigma-Aldrich #B9934, MO, USA) for 30 min at the room temperature and washed three times in PBS. Images were acquired using a Leica TCS SP8 confocal microscope (Leica, Germany) equipped with a 63 × /1.4 numerical aperture oil-immersion objective. For each condition, 10–15 images were acquired from at least five organoids from three independent experiments (cultures). For quantification of tau/p-tau, APP, and ubiquitin immunoreactivity, the mean fluorescence intensity of the whole field was measured using Image J. Quantification of cleaved caspase-3-positive neurons was performed using Fiji distribution of ImageJ from confocal z-stacks (the number of cleaved caspase-3-positive cell was normalized on total number of β-TUBIII/DAPI positive cells from the whole field). For analyses of amyloid-like plaque size, the measurement of the particle area was performed using the ImageJ “threshold/analyze particle” plugin.

### Calcium imaging

For cytosolic calcium imaging, neurons were loaded with 1 μM Fluo-4-AM (Invitrogen, MA, USA) for 30 min at 37 °C. After loading, cells were washed twice with Krebs-Ringer-HEPES (20 mM HEPES, 135 mM NaCl, 5 mM KCl, 1 mM MgSO4, 1 mM CaCl2, and 5.5 mM glucose, pH 7.4) (KRH) buffer and imaged using a Leica TCS SP8 confocal microscope (Leica, Germany) equipped with a 63 × /1.4 numerical aperture oil-immersion objective. After 1 min of equilibration time (baseline), neurons were exposed to 0.6 mM KCl. Fluo-4 AM was excited at 488 nm and the emission was collected at 510 nm. Images were captured every 25 s. All images were processed using Image J software. Data were normalized to the baseline.

### Measurement of mitochondrial membrane potential

For measurement of the mitochondrial membrane potential, iPSC-derived neurons were incubated with 100 nM Tetramethylrhodamine Methylester Perchlorate (TMRM) and 100 nM MitoTracker Green (both from Invitrogen, MA, USA) in a KRH buffer for 30 min at 37 °C and then imaged using a Leica TCS SP8 confocal microscope (Leica, Germany) with a 63 × /1.4 numerical aperture oil-immersion objective. The 568 nm laser line was used to excite TMRM and the 488 nm laser line to excite Mito-Tracker Green. Emitted fluorescence was measured above 574 nm for TMRM and between 490 and 530 nm for MitoTracker Green. Z-stacks were acquired for calculations of the mitochondrial membrane potential. Image J was used to quantify TMRM intensity in individual MitoTracker-Green-positive mitochondria. Basal TMRM levels were taken as 100% and the remaining TMRM fluorescence in the mitochondria after complete depolarization by FCCP was regarded as background. Intensities were corrected for background subtraction. Data were obtained from *n* > 40 cells from three independent experiments.

### Measurement of mitochondrial reactive oxygen species (mtROS)

For the measurement of mtROS production of iPSC-derived neurons by flow cytometry, cells were preincubated with N2 medium for 48 h. Then, 1 × 10^6^ cells were washed once with HBSS, incubated with 5 μM of the superoxide indicator MitoSOX Red (Invitrogen, MA, USA) for 30 min at 37 °C, washed twice with HBSS, dissociated using trypsin (Invitrogen, MA, USA), and resuspended in 200 μL of HBSS and 1% BSA. The cytofluorimetric analysis was performed using MACSQuant Analyzer 10 (Miltenyi Biotec, Germany).

### Seahorse XF_p_ metabolic flux analysis

The oxygen consumption rate was analyzed using an XFp Extracellular Flux Analyzer (Agilent, CA, USA). iPSC-derived neurons were plated on XFp microplates (Agilent, CA, USA) at a density of 70,000 per well and grown in N2 medium for 48 h before the experiment. Measurement of the neuronal oxidative consumption rate was performed in a freshly prepared medium consisting of phenol-free DMEM, 1 mM sodium pyruvate, 2 mM glutamine, and 10 mM glucose with the pH adjusted to 7.4. Mitochondrial function was evaluated at baseline levels and after the subsequent injection of 10 μM oligomycin, 10 μM carbonyl cyanide p-trifluoromethoxyphenylhydrazone, and 1 μM antimycin A/1 μM rotenone (all Sigma-Aldrich, MO, USA). Three measurements, lasting 5 min each, were taken for each condition. The measured values were normalized on protein concentration measured by BCA.

### Quantitative RT-PCR

mRNA was isolated using an RNA isolation kit (Qiagen, Germany). Following the reverse transcription reaction using the QuantiTect Reverse Transcription Kit, a quantitative PCR reaction was performed using SYBR Green (all Qiagen, Germany) and monitored with a Viia7 Real-Time PCR system (Applied Biosystems, CA, USA). The expression level of each gene was normalized to the housekeeping gene ribosomal protein large P0 (Rplp0). Fold changes in gene expression were calculated using the 2^–DDCT^ method, based on biological reference samples and housekeeping genes for normalization.

**Table:** 

qPCR primers (5′–3′)		
*GAPDH*	Fw	AGGGGAGATTCAGTCTGG
	Rv	CGACCACTTTGTCAAGCT
*HSP60*	Fw	TGACCCAACAAAGGTTGTGA
	Rv	CATACCACCTCCCATTCCAC
*LONP1*	Fw	CCCGCGCTTTATCAAGATT
	Rv	AGAAAGACGCCGACATAAGG
*ATF4*	Fw	GTCCCTCCAACAACAGCAAG
	Rv	CTATACCCAACAGGGCATCC
*DDIT3*	Fw	AGCCAAAATCAGAGCTGGAA
	Rv	TGGATCAGTCTGGAAAAGCA
*ERO1A*	Fw	AGCGGCACAGAGGTGCT
	Rv	TGTAGTCTTGGGAAAAGCCTG
*RPLP0*	Fw	CCTCATATCCGGGGGAATGTG
	Rv	GCAGCAGCTGGCACCTTATTG
*HSPA9*	Fw	GGAAGCTGCTGAAAAGGCTA
	Rv	CTTGGGTCCAGAAGAATCCA
*CLPP*	Fw	CTCTTCCTGCAATCCGAGAG
	Rv	GGATGTACTGCATCGTGTCG
*16S* (mtDNA)	Fw	GCCTTC CCCCGTAAATGATA
	Rv	TTATGCGATTACCGGGCTCT
*PITRM1*	Fw	ATCTGTTCCCGAGCTGTTCC
	Rv	GAAAGGCTCTCTGCACGGAT
APP	Fw	TGGAGGTACCCACTGATGGT
	Rv	TTGTCAGGAACGAGAAGGGC

### Mitochondrial isolation

Mitochondrial isolation was performed using a Qproteome^®^ Mitochondria Isolation Kit (Qiagen, Germany) according to the manufacturer’s protocol.

### Western blot

Proteins were extracted using tris-buffered Saline (TBS) with a 0.5% NP40 protein extraction buffer containing protease and phosphatase inhibitors (Roche, Switzerland), on ice, following centrifugation at 14,000 rpm and 4 °C for 15 min. The protein concentration of the supernatant was determined by BCA (Pierce, WI, USA). In total, 15–30 μg of the protein lysate was loaded on polyacrylamide gel (the density ranged from 7.5 to 15%, depending on the respective protein molecular weight) and transferred on a PVDF membrane (Millipore, MA, USA). Blots were blocked with 5% milk powder or 5% BSA in TBS + 0.1% Tween-20 (TBST) and incubated with primary antibodies in milk or BSA blocking solution overnight at 4 °C. This step was followed by incubation with corresponding HRP-conjugated secondary antibodies (Sigma-Aldrich, MO, USA) for 1 h at the room temperature. Visualization of proteins was carried out using Amersham ECL Western Blotting Detection Reagent and Amersham Hyperfilm (both GE Healthcare, IL, USA). Densitometric analysis of proteins was performed by ImageJ software. Primary antibodies included rabbit anti-LC3B (1:500, Cell Signaling Technologies #2775, MA, USA), mouse anti-APP 6E10 (Abeta 1-16) (1:1000, BioLegend #803004, CA, USA), rabbit anti-PITRM1 (1:1000, Atlas Antibodies #HPA006753, Sweden), rabbit anti-Frataxin (1:1000, Abcam #ab175402, UK), mouse total OXPHOS human WB antibody cocktail (1:1000, Abcam #ab110411, UK), mouse anti-ubiquitin (1:5000, Millipore #MAB1510, MA, USA), mouse anti-HSPA9/GRP 75 (1:3000, Santa Cruz Biotechnology #sc-133137, TX, USA), mouse anti-HSP60 (1:3000, Santa Cruz Biotechnology #sc-271215, TX, USA), rabbit anti-LONP1 (1:3000, Proteintech #15440-1-AP, IL, USA), mouse anti-tau (1:1000, HT7, Thermo Fisher Scientific #MN1000, MA, USA), mouse anti phospho-tau PHF-6 (Thr231) (1:1000, Thermo Fisher Scientific #35-5200, MA, USA), mouse anti phospho-tau AT8 (Ser202, Thr205) (1:1000, Thermo Fisher Scientific #MN1020, MA, USA), mouse anti phospho-tau Thr181 (1:1000, Thermo Fisher Scientific #MN1050, MA, USA), mouse anti-β-Actin (1:20000, Sigma-Aldrich #A5441, MO, USA), and mouse anti-VDAC1 (1:1000, Santa Cruz Biotechnology #sc-390996, TX, USA).

### Autophagy studies

Where indicated, cells were treated with NH_4_Cl (20 mM) and leupeptin (200 μM) (EMD, Millipore, MA, USA) for 4 h. Light chain type 3 protein (LC3-II) and LC-I levels were quantified by densitometry and normalized to β-actin. LC3 flux was quantified by dividing the levels of LC3-II after treatment with lysosomal inhibitor for 4 h by the levels of LC3-II without treatment.

### Immunofluorescence

Cells were fixed in 4% paraformaldehyde (PFA) in PBS (w/v) for 10 min, rinsed with PBS, and blocked by 10% normal goat or donkey serum (NGS/NDS) in PBST (PBS + 0.1% Triton X-100) for 60 min. Cells were then incubated with primary antibodies in 10% NGS/NDS in PBST overnight at 4 °C following 1 h of incubation at 24 °C with the appropriate Alexa 488/568 coupled secondary antibodies (1:1000, Invitrogen, MA, USA). Cell nuclei were stained with DAPI; final images were acquired using a Leica TCS SP8 confocal microscope (Leica, Germany) and analyzed using Fiji software. Primary antibodies included rabbit anti-LC3B (1:500, Cell Signaling Technologies #2775, MA, USA), rabbit anti-TBR1 (1:500, Abcam #ab31940, UK), and mouse anti-β-III-tubulin (1:1000, BioLegend, CA, USA. Previously Covance PRB-435P). For live imaging of Aβ in neuronal cultures, cells were incubated with 100 nM BTA-1 and 1 μM NeuroFluor™ NeuO (Invitrogen, MA, USA) for 20 min. The LC3 particle number in β-III-tubulin-positive cells was quantified with the “analyse particles” plug-in in ImageJ (NIH). Quantification was carried out on at least 50 cells per condition, from three independent experiments.

### LDH assay

The LDH assay (Promega, WI, USA) was performed as per the manufacturer’s instructions.

### Amyloid β species measurement

For amyloid β species measurement in iPSC-cortical neurons, 10^6^ cells were plated into one well of a 12-well plate; cell supernatant was collected after 5 days, snap-frozen, and stored at −80 °C until analysis. A cell pellet was collected and lysed to determine the protein concentration and used for value normalization. Conditioned medium from at least three technical replicates was collected in each experiment. For amyloid β species measurement in cerebral organoids, individual organoids were replated into low-attachment 96-well plates on an orbital shaker. Supernatant was collected after 5 days, snap-frozen in liquid nitrogen, and stored at −80 °C until analysis. After the collection of the supernatant, individual organoids were lysed and the protein concentration was measured with BCA for value normalization. The concentrations of Aβ40 (Aβx–40) and Aβ42 (Aβx–42) in the samples were measured on a Sector Imager 6000 using an electro chemiluminescence-based immunoassay, V-PLEX Aβ Peptide Panel 1 (6E10) Kits (Meso Scale Discovery, MD, USA) according to the manufacturer’s instructions. All samples were thawed on ice and diluted 1:2 in a buffer (Diluent 35, Meso Scale Discovery, MD, USA) before incubation. A neural differentiation medium without B27/N2 supplements was used as the negative control for each measurement. Human CSF samples were used as internal references on each plate. At least eight organoids were used for each measurement. Every sample was tested in duplicate (and excluded if the coefficient of variance was above 20%). Data analysis was run using the MSD DISCOVERY WORKBENCH software, version 2.0.

### ELISA

Individual cerebral organoids were homogenized in an icecold RIPA buffer containing protease and phosphatase inhibitors (Roche, Switzerland), following centrifugation at 14,000 rpm and 4 °C for 10 min. The protein concentration of the supernatant was determined by BCA (Pierce, WI, USA). Total and phospho-tau levels, as well as IL-1β and TNF-α levels, were assessed, in equal protein amounts, using ELISA assay kits (total-tau #KHB0041 and phoshotau #KHO0631, both Invitrogen (MA, USA); IL-1β #DY201 and TNF-α #DY210, both DuoSet ELISA, R&D Systems, MN, USA), according to the manufacturer’s instructions.

### 10X Genomics single-cell RNA sequencing

Cerebral organoids were dissociated into a single-cell suspension using a Worthington Papain Dissociation System kit (Worthington Biochemical, OH, USA), as per the manufacturer’s instructions. Cerebral organoids were minced and incubated with a 2.5 Papain/DNAase solution on an orbital shaker for 1 h at 37 °C. Organoid suspensions were intermittently triturated (30 times every 30 min) using 1 ml low-attachment pipette tips. Cell suspensions underwent several filtering (40, 30 μm) and washing steps with HBSS. The final solution was centrifuged 300 *g* per 7 min and the cell pellet was resuspended in PBS-0.5% BSA and filtered using a 30 μm strainer. The final preparation contained over 95% live-cells at a concentration of 1000 cells/μL. Single-cell suspension concentration and cell viability were determined by microscopy using trypan blue staining (0.2%) and by automatic cell counting (DeNovix CellDrop, DE, USA) using an AO/PI viability assay (DeNovix, DE, USA). scRNA-seq libraries were generated using the 10X Chromium Next gel beads-in-emulsion (GEM) Single Cell 3’ Reagent Kit v3.1 according to the manufacturer’s instructions. Approximately 18,000 cells per sample were loaded on the Chromium Next GEM Chip G, which was subsequently run on the Chromium Controller (10X Genomics, CA, USA) to partition cells into GEMs. Cell lysis and reverse transcription of poly-adenylated mRNA occurred within the GEMs and resulted in cDNA with GEM-specific barcodes and transcript-specific unique molecular identifiers (UMIs). After the breaking of the emulsion, cDNA was amplified (11 cycles), enzymatically fragmented, end-repaired, extended with 3′ A-overhangs, and ligated to adapters. P5 and P7 sequences, as well as sample indices (Chromium i7 Multiplex kit, 10X Genomics, CA, USA), were added during the final PCR amplification step. The fragment size of the final libraries was determined using the Bioanalyzer High-Sensitivity DNA Kit (Agilent, CA, USA). Their concentration was determined using the Qubit dsDNA HS Assay Kit (Thermo Fisher Scientific, MA, USA). scRNA libraries were pooled and paired-end-sequenced on the Illumina NovaSeq 6000 platform.

### Single-cell sequencing data analysis

Samples were demultiplexed and the 10X Genomics pipeline cellranger count was run to generate filtered gene-barcode matrices (the number of UMIs associated with a gene and a cellular barcode). Filtered gene-barcode matrices were used as input for downstream analysis using the R package Seurat [16, 17]. Seurat objects were merged and potential low-quality cells (UMI < 2500, detected genes <500) and cells with UMI counts over 15,000 were removed. Each dataset was normalized by dividing the gene count for each cell by the total counts of that cell. The results were multiplied by 10,000 (scale factor), natural-log-transformed, and used as input to determine the cell cycle scores (Seurat CellCycleScoring) for each cell and to visualize gene expression of the marker gene. Subsequently, each dataset was SCtransform normalized and prepared for sample integration by performing canonical correlation analysis to identify the greatest sources of variation conserved across all samples, followed by anchor identification and filtering. After integration of the samples, principle components (PCs) were determined and, using the first 40 PCs, cells were clustered using a K-nearest neighbor graph with a clustering resolution of 0.8, resulting in 26 clusters. Cell clusters were visualized using UMAP. Conserved markers were determined across all samples for each cluster (FindConservedMarkersfunction) using the default settings (min.pct=0.25, logfc.threshold=0.25) and used for assigning the cell type to cell clusters. Differently expressed genes between conditions in each cluster were determined using the FindMarkers function with the Wilcoxon Rank Sum test (min.pct=0.1, logfc.threshold=0.1) and *p* value adjustment by Bonferroni correction based on the total number of genes in the dataset. Pathway analysis of differentially expressed genes (DEGs) was performed using Ingenuity pathway analysis (IPA^©^ 2000–2020, Ingenuity^®^ Systems, www.ingenuity.com). scRNA-seq data are available at NCBI GEO (GEO accession number GSE147047).

### Statistical analysis

The Statistical Package GraphPad Prism versions 7 and 8.3 (GraphPad Software) was used to analyze the data. Statistical significance was evaluated using a two-tailed Student’s *t* test. Data are expressed as mean + S.E.M.

## Results

### Mitochondrial dysfunction in PITRM1-knockout iPSC-derived neurons

**Fig. 1:**
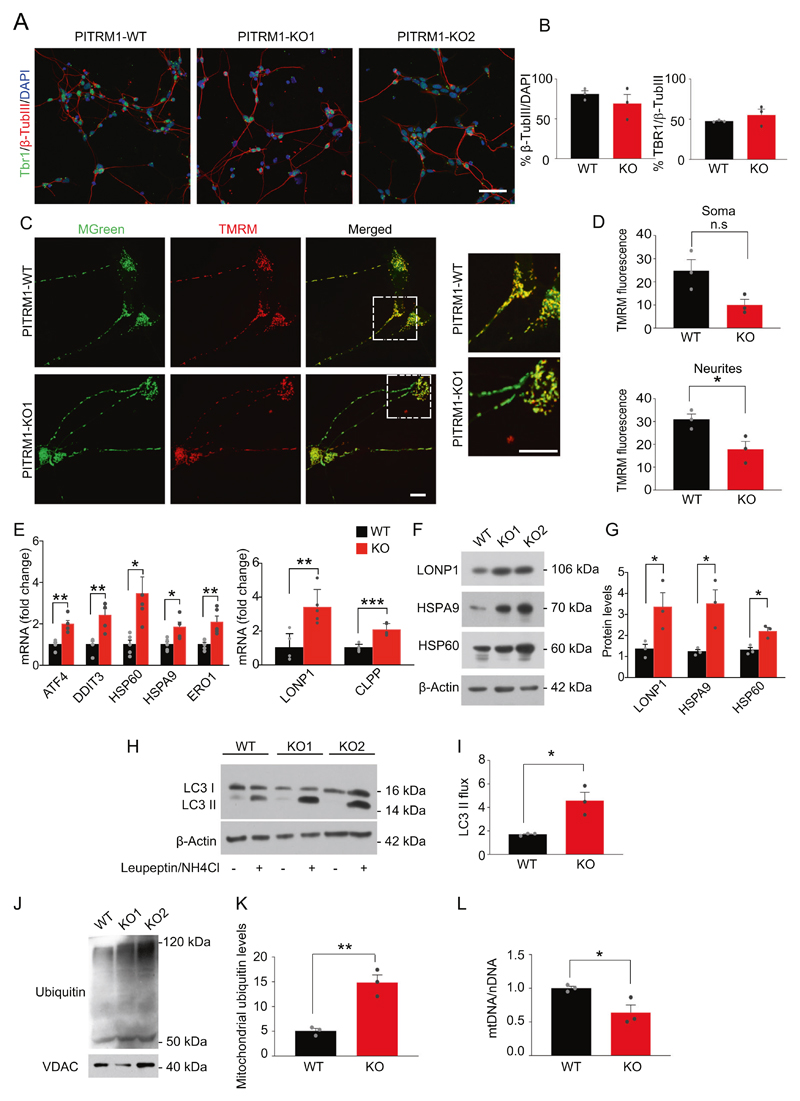
PITRM1^−/−^ iPSC-derived neurons show the induction of UPRmt and enhanced mitophagy. Control PITRM1^+/+^ (WT) and isogenic PITRM1^−/−^ (KO) iPSCs were differentiated into cortical neurons. **a** Immunostaining of indicated differentiated iPSC cultures at DIV 35. Cells were stained for TBR1 (green) and β-III-tubulin (β-TUBIII, red). Nuclei were counterstained with DAPI (blue). Scale bar,50 μm. **b** Quantification of β-TUBIII/DAPI and TBR1/β-TUBIII-positive cells in differentiated iPSC cultures at DIV 35 (mean + SEM; *n* = 3). **c** Isogenic PITRM1^+/+^ and PITRM1^−/−^ iPSC-derived neurons were stained with tetramethylrhodamine methylester (TMRM) and MitoTracker Green (MGreen) fluorescent dyes to determine mitochondrial membrane potential and mitochondrial mass, respectively. Representative images are shown. Scale bar, 10 μm. **d** Mitochondrial membrane potential (TMRM fluorescence) in neuronal soma and neurites (mean + SEM; *p < 0.05, two-tailed *t* test, *n* = 3). **e** Gene expression levels of mitochondrial stress response genes in PITRM1^+/+^ and PITRM1^−/−^ iPSC-derived cortical neurons (mean + SEM; *p < 0.05, **p < 0.01, ***p < 0.001, two-tailed *t* test, *n* = 5). **f**, **g** Representative western blots of the mitochondrial chaperones HSPA9 and HSP60 and the mitochondrial protease LONP1 in PITRM1^+/+^ and PITRM1^−/−^ iPSC-derived cortical neurons. Quantification of protein levels relative to the loading control is shown in (**g**) (mean + SEM; *p < 0.05, two-tailed *t* test, *n* = 3). **h** Western blot analysis for LC3 in PITRM1^+/+^ and PITRM1^−/−^ iPSC-derived neuronal cultures, untreated (—) or treated with 200 μM leupeptin and 20 mM NH_4_Cl for 4 h (+). **i** Quantification of LC3 flux normalized to WT (mean + SEM; *p < 0.05, two-tailed *t* test, *n* = 3). **j** Representative western blot of isolated mitochondria from PITRM1^+/+^ and PITRM1^−/−^ iPSC-derived neurons probed against ubiquitin and VDAC as the loading control. **k** Quantification of mitochondrial protein ubiquitination levels in PITRM1^+/+^ and PITRM1^−/−^ iPSC-derived neurons (mean + SEM; **p < 0.01, two-tailed *t* test, *n* = 3). **I** mtDNA content was measured as the mitochondrial (*16S*) to nuclear (*RPLP0*) DNA ratio by qRT-PCR (mean + SEM; *p < 0.05, two-tailed *t* test, *n* = 3).

To overcome the limitation of the embryonic lethality previously observed in PITRM1-knockout mice [2] and examine the mechanistic link between PITRM1 deficiency and neurodegeneration in a model that more closely resembles human disease, we generated PITRM1-knockout (PITRM1^−/−^) human iPSCs using CRISPR/Cas9 endonuclease-mediated gene editing. Appropriate sgRNAs targeting exon 3 and exon 4 were designed to introduce a frameshift deletion resulting in the complete knockout of the PITRM1 protein (Supplementary Fig. 1a). Several homozygous clones were generated and two fully characterized clones were selected and used for further analysis (Supplementary Fig. 1b-e). To address the impact of PITRM1 on the function of human neurons, we differentiated PITRM1^+/+^ and PITRM1^−/−^ iPSCs into neuronal cultures that were enriched for cortical neurons; we assessed the neuronal cultures at 35 and 65 days in vitro (DIV). Western blot analysis confirmed the complete absence of PITRM1 protein in differentiated PITRM1^−/−^ iPSCs (Supplementary Fig. 1d, e). Both PITRM1^+/+^ and PITRM1^−/−^ iPSCs efficiently generated cortical neurons without significant differences (Fig. 1a, b). Furthermore, PITRM1^+/+^ and PITRM1^−/−^ showed a similar increase in cytosolic calcium after KCl stimulation (Supplementary Fig. 1f, g). No overt cell death was observed in neuronal cultures, as assessed by LDH assay at 35 and 65 DIV (Supplementary Fig. 1h). Previous work showed that PITRM1 deficiency leads to the accumulation of non-degraded MTS sequences that, in turn, impair the processing of presequence proteins by the peptidase MPP [9]. Because mitochondrial peptidases are also involved in the maturation of the human frataxin precursor [18, 19], we examined frataxin maturation by immunoblotting. A decreased ratio of processed, mature to immature frataxin was detected in PITRM1^−/−^ iPSC-derived NPCs and neurons (Supplementary Fig. 1i, j), indicating the impaired function of MPP and defects of mitochondrial presequence processing. Because MTS peptides can bind to the membrane and perturb the mitochondrial electrochemical gradient, we evaluated the effect of PITRM1 deficiency on mitochondrial membrane potential and respiratory oxidative activity. Mitochondrial membrane potential was reduced in PITRM1^−/−^ neurons compared with isogenic PITRM1^+/+^neurons (Fig. 1c, d). Interestingly, this decrease was more evident in neurites than in soma (Fig. 1c, d). However, mtROS were not significantly altered in PITRM1^−/−^ neurons (Supplementary Fig. 2a). Similarly, no significant difference in respiratory oxidative activity was observed between PITRM1^+/+^ and PITRM1^−/−^ neurons (Supplementary Fig. 2b). Western blot analysis revealed a significant increase in the level of Complex II respiratory chain complex subunits in PITRM1^−/−^ neurons (Supplementary Fig. 2c, d). However, no significant difference for the levels of all other respiratory chain complex subunits was detected in PITRM1^−/−^ neurons (Supplementary Fig. 2c, d).

### PITRM1^−/−^ iPSC-derived cortical neurons show the induction of mitochondrial stress response and enhanced mitophagy

Mitochondrial stress response has been identified as a common signature in several neurodegenerative as well as primary mitochondrial diseases [20–23]. Thus, we examined the expression levels of genes involved in mitochondrial unfolded protein response (UPR^mt^) and, more generally, in the mitochondrial integrated stress response pathway (mtISR). PITRM1^−/−^ iPSC-derived cortical neurons exhibited a significant induction of UPR^mt^/mtISR transcripts (*ATF4, DDIT3, HSP60, HSPA9, ERO1*) (Fig. 1e). Moreover, gene expression of the mitochondrial proteases, *LONP1* and *CLPP*, was significantly upregulated in PITRM1^−/−^ neurons as compared with the controls (Fig. 1e). In line with the gene expression data, we found an increase in the protein expression of the chaperones HSPA9 and HSP60 and the mitochondrial protease LONP1 (Fig. 1f, g). These data suggest that PITRM1 deficiency leads to a strong upregulation of the ISR pathway. Because the ISR has been shown to activate autophagy [24, 25], we assessed the autophagosome content by immunostaining for endogenous LC3, a marker of autophagosomes. Our analysis revealed a significant decrease of LC3-positive vesicles in PITRM1^−/−^ neurons compared with isogenic PITRM1^+/+^ neurons (Supplementary Fig. 2e, f). These results were confirmed by western blot, showing decreased basal levels of LC3-II in PITRM1^−/−^ neurons (Fig. 1h). However, inhibition of lysosomal degradation by leupeptin and ammonium chloride revealed that the autophagic flux was significantly increased in PITRM1^−/−^ neurons, thus confirming autophagy activation (Fig. 1h, i). To assess whether the observed increase in autophagic flux leads to an enhanced turnover rate of mitochondria by autophagy, we purified mitochondria from PITRM1^+/+^ and PITRM1^−/−^ iPSC-derived cortical neurons. Immunoblotting of purified mitochondria showed increased ubiquitination of PITRM1^−/−^ neurons as compared with PITRM1^+/+^ neurons (Fig. 1j, k), suggesting their targeting to lysosomes and increased mitochondrial clearance [26]. In line with these results, mitochondrial content was lower in PITRM1^−/−^ neurons, as shown by a reduced mitochondrial-to-nuclear-DNA ratio (Fig. 1l). Taken together, these results suggest that the loss of PITRM1 function enhances mitochondrial clearance.

### PITRM1^−/−^ iPSC-derived neurons show the accumulation of APP and an increase in extracellular Aβ peptides levels

**Fig. 2:**
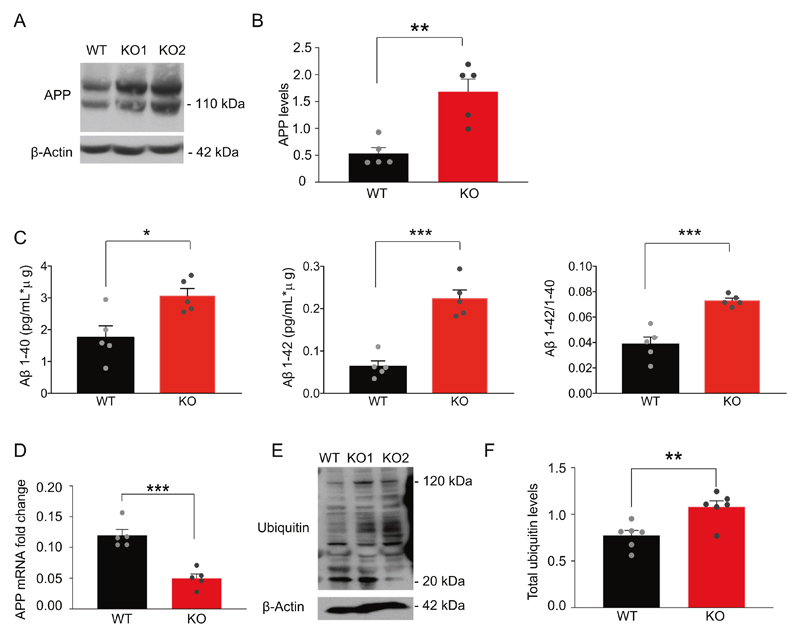
PITRM1^−/−^ iPSC-derived neurons show the accumulation of APP and increased levels of Aβ peptides. **a** Representative western blots of APP in PITRM1^+/+^ and PITRM1^−/−^ iPSC-derived cortical neurons. **b** Quantification of APP levels normalized to the loading control (mean + SEM; **p < 0.01, two-tailed *t* test, *n* = 5). **c** Quantification of Aβ species and the Aβ42/Aβ40 ratio in the supernatant of PITRM1^+/+^ and PITRM1^−/−^ iPSC-derived cortical neurons at DIV 35, as performed by Meso Scale immunoassay (mean + SEM; *p < 0.05, ***p < 0.001, two-tailed *t* test, *n* = 5). **d** Gene expression levels of APP in PITRM1^+/+^ and PITRM1^−/−^ iPSC-derived cortical neurons (mean + SEM; ***p < 0.001, two-tailed *t* test, *n* = 5). **e** Representative western blot of PITRM1^+/+^ and PITRM1^−/−^ iPSC-derived neurons of total ubiquitinated protein levels. **f** Quantification of the protein ubi-quitination level in PITRM1^+/+^ and PITRM1^−/−^ iPSC-derived neurons (mean + SEM; **p < 0.01, two-tailed *t* test, *n* = 6).

To examine the impact of PITRM1 activity on Aβ pathology, we first assessed the levels of APP by western blot. APP protein levels were found to be significantly increased in PITRM1^−/−^ neurons as compared with PITRM1^+/+^ neurons (Fig. 2a, b). Next, supernatant from PITRM1^+/+^ and PITRM1^−/−^ cortical neurons was analyzed using the MesoScale Discovery immunoassay for human Aβ peptides. PITRM1^−/−^ neurons showed significantly higher levels of Aβ40 and Aβ42 peptides as well as an increased Aβ42/Aβ40 ratio compared with control samples (Fig. 2c). Remarkably, Aβ peptides were not detected in mitochondrial extracts from PITRM1-deficient neurons. Next, we explored the mechanisms that link the loss of PITRM1 function with the alteration of APP metabolism. We found a significant decrease of APP mRNA levels in PITRM1^−/−^ neurons (Fig. 2d). Because UPR^mt^ may have an impact on ubiquitin-dependent protein turnover [27], we examined the levels of ubiquitinated proteins by western blot. We observed that PITRM1^−/−^ display increased levels of ubi-quitinated proteins (Fig. 2e, f), suggesting defects in cellular proteostasis. Despite APP accumulation and an increased Aβ42/Aβ40 ratio, 2D iPSC-derived neuronal cultures did not show Aβ aggregates (Supplementary Fig. 2g). We found an increase in both phosphorylated tau (pThr231, pSer202 + Thr205, and pThr181) and total tau in PITRM1^−/−^ neurons (Supplementary Fig. 2h). However, the p-tau/total tau ratio was not significantly altered (Supplementary Fig. 2h, i).

### scRNA-seq of PITRM1^−/−^ cerebral organoids reveals a cell-type-specific impact

**Fig. 3:**
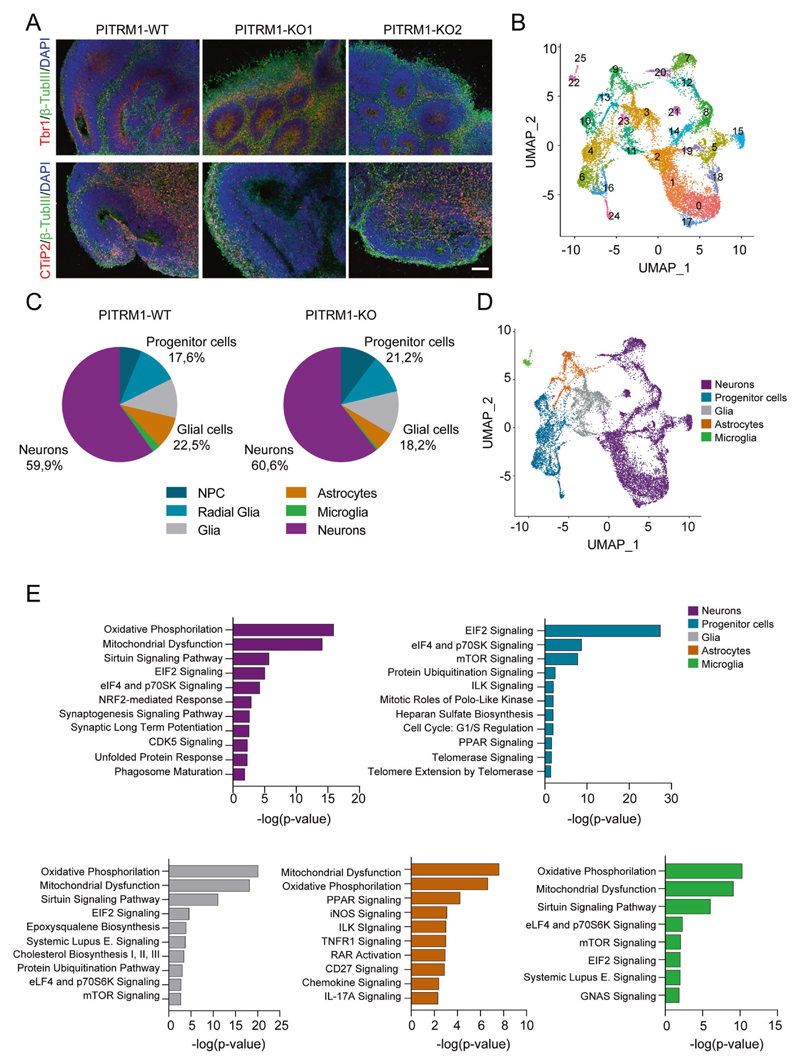
scRNA-seq of cerebral organoids reveals a cell-type-specific impact of PITRM1. **a** Generation and characterization of cerebral organoids from PITRM1^+/+^ and PITRM1^−/−^ iPSCs. Immunostaining for β-TUBIII (green), TBR1 (red), and CTIP2 (red) of 2-month-old cerebral organoids. Cell nuclei were counterstained with DAPI (blue). Scale bar, 100 μm. **b** UMAP dimensionality reduction plot showing the unsupervised clustering of PITRM1^+/+^ and PITRM1^−/−^ cerebral organoids. Total *n* = 15,183 cells. **c** Pie charts showing the frequency of major cell clusters across the genotypes. Cell clusters were determined by expression of specific markers. **d** UMAP dimensionality reduction plot showing five distinguished clusters with cell-type identities as determined by the expression of specific markers. **e** Ingenuity pathway analysis (IPA) of DEG in neurons (cluster 1), progenitor cells (cluster 24), glial cells (cluster 3), astrocytes (cluster 13), and microglia (cluster 22).

To further investigate the mechanisms of PITRM1 neurotoxicity in a model that better resembles the human disease, we developed cerebral organoids from PITRM1^+/+^ and PITRM1^−/−^ iPSCs and cultured them over a broad time span (1–6 months). Cerebral organoids derived from PITRM1^+/+^ and PITRM1^−/−^ iPSCs displayed similar sizes and cortical layering characteristics (Fig. 3a and Supplementary Fig. 3a). To comprehensively characterize the cell types present in cerebral organoids and gain insight into cell-type-specific mechanisms, we transcriptionally profiled 2-month-old cerebral organoids using 10X Genomics Chromium Single Cell RNA Sequencing. Using an unsupervised cell-clustering algorithm, we identified 26 separate cell clusters. Individual clusters contained between 32 and 1712 cells and were defined by the first 40 principal components (Fig. 3b). For specific gene expression comparisons, these clusters were manually assigned based on the expression of known cell-type-specific markers (Fig. 3c, Supplementary Fig. 3b, c and Supplementary Table 1) to progenitor cells, including NPC (*TOP2A, MKI67, CENPF, UBE2C*) and radial glia (*SLC1A3, SOX2, GLI3, HES1, PAX6, SOX9*) (clusters 4, 6, 10, 16, and 24), neurons (*STMN2, DCX, RELN, NEUROD2, NEUROD6*) (clusters 0, 1, 2, 5, 7, 8, 12, 14, 15, 17, 18, 19, 20, and 21), astrocytes (*GJA1, S100B*) (clusters 9 and 13), microglia (*TMEM119, OLFML3*) (clusters 22 and 25), and additional glial cell types not contained in these groups (*CRYAB, OLIG1, APOE, HLA-B, CEBPB*) (clusters 3, 11, and 23) (Fig. 3c, d). Among the neuronal clusters, we further identified layer I cortical neurons (clusters 18 and 21), upper cortical layer neurons (clusters 0, 1, 2, and 17), and dopaminergic neurons (cluster 15). For both genotypes, PITRM1^+/+^ and PITRM1^−/−^, all clusters were similarly represented, with 60% of the identified single cells expressing neuronal lineage markers and almost 20% expressing glial cell markers (Fig. 3c, d). To identify cell-type-specific mechanistic pathways associated with DEG sets between isogenic PITRM1^+/+^ and PITRM1^−/−^ cerebral organoids, we performed pathway analysis in the different clusters. Canonical pathway analysis revealed that mitochondrial function, oxidative phosphorylation, and the sirtuin pathway were common dysregulated pathways among neurons (cluster 1), glial cells (cluster 3), astrocytes (cluster 13), and microglia (cluster 22) (Fig. 3e and Supplementary Table 2). Interestingly, synaptogenesis and long-term potentiation emerged as differentially dysregulated pathways in neurons, whereas inflammatory pathways (including iNOS, PPAR signaling TNFR1, RAR activation, chemokine, and IL-17A signaling pathways) were significantly dysregulated in astrocytes. In line with an impact of PITRM1 deficiency on immune pathways, we found a significant increase of the inflammatory cytokine TNF-α in PITRM1^−/−^ cerebral organoids (Supplementary Fig. 3d). In addition, we found a non-significant increase in IL-1β levels (Supplementary Fig. 3d).

### PITRM1^−/−^ cerebral organoids exhibit the main features of AD pathology and the induction of mitochondrial stress response

**Fig. 4:**
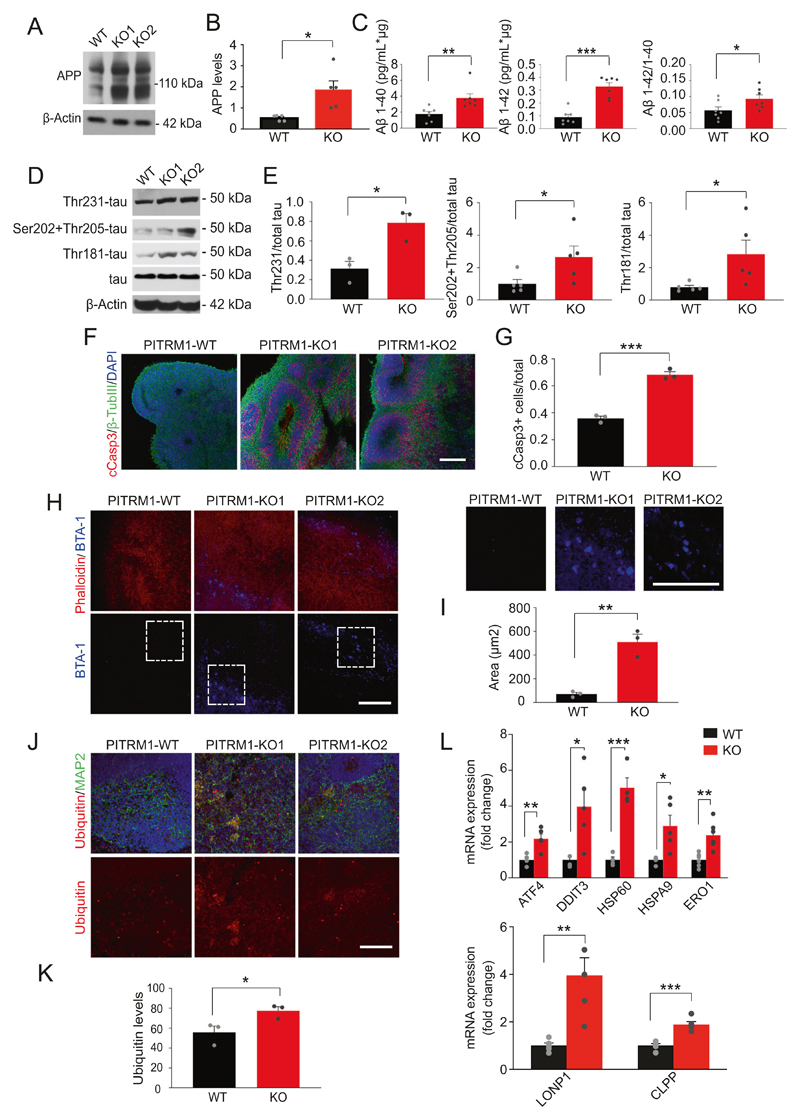
PITRM1^−/−^ cerebral organoids display the main pathological features of AD pathology and the induction of the mitochondrial stress response. **a** Western blot of APP in 2-month-old PITRM1^+/+^ and PITRM1^−/−^ cerebral organoids. **b** Quantification of APP protein levels in cerebral organoids (mean + SEM; *p < 0.05, two-tailed *t* test, *n* = 5). **c** Quantification of Aβ species in the supernatant of PITRM1^+/+^ and PITRM1^−/−^ iPSC-derived cerebral organoids, as performed by Meso Scale immunoassay (mean + SEM; *p < 0.05, **p < 0.01, ***p < 0.0001, two-tailed *t* test, *n* = 7). **d** Representative western blots of phospho-tau (Thr231, Ser202 and Thr205, Thr181 phosphorylation sites) and total tau in PITRM1^+/+^ and PITRM1^−/−^ cerebral organoids; total tau and β-Actin were used as the loading controls. **e** Quantification of phospho-tau protein levels in cerebral organoids relative to the loading control total tau/β-Actin (mean + SEM; *p < 0.05, two-tailed *t* test, *n* = 3-5). **f** Immunostaining for β-TUBIII (green) and cleaved caspase-3 (cCASP3, red) in PITRM1^+/+^ and PITRM1^−/−^ 2-month-old cerebral organoids. Cell nuclei were counterstained with DAPI (blue). Scale bar, 100 μm. **g** Analysis of the ratio of cCASP3-positive cells relative to the total number of β-TUBIII/DAPI cells in 2-month-old cerebral organoids (mean + SEM; ***p < 0.001, two-tailed *t* test, *n* = 3). **h** Phalloidin (red) and BTA-1 (blue) staining in cerebral organoids. Representative confocal images are shown. Scale bars, 100 μm. **i** Quantification of the size of BTA-1-positive areas in cerebral organoids (mean + SEM; **p < 0.01, two-tailed *t* test, *n* = 3). **j** MAP2 (green) and ubiquitin (red) immunostaining in cerebral organoids. Representative confocal images are shown. Cell nuclei were counterstained with DAPI (blue). Scale bars, 100 μm. **k** Quantification of ubiquitin mean fluorescent intensity (mean + SEM; *p < 0.05, two-tailed *t* test, *n* = 3). **l** Gene expression levels of mitochondrial stress response genes in PITRM1^+/+^ and PITRM1^−/−^ cerebral organoids (mean + SEM; *p < 0.05, **p < 0.01, ***p < 0.001, two-tailed *t* test, *n* = 4-6).

Next, we examined whether PITRM1^−/−^ cerebral organoids develop AD-like neurodegenerative features. Western blotting revealed increased APP levels in 2-month-old PITRM1^−/−^ cerebral organoids (Fig. 4a, b). Furthermore, immunoassay measurements showed a higher Aβ40, Aβ42, and Aβ42/Aβ40 ratio in PITRM1^−/−^ cerebral organoids compared with controls (Fig. 4c). Western blot analysis revealed tau hyperphosphorylation in 2-month-old PITRM1^−/−^ cerebral organoids compared with PITRM1^+/+^ organoids (Fig. 4d, e). Similarly, immunofluorescence staining confirmed increased APP and phospho-tau levels in PITRM1^−/−^ organoids compared with PITRM1^−/−^ controls starting at 2 months (Supplementary Fig. 4a, b). No further increase of tau hyperphosphorylation was observed at later time points (Supplementary Fig. 4c). Next, we stained 1-, 2-, and 6-month-old PITRM1^+/+^ and PITRM1^−/−^ cerebral organoids, for cleaved caspase-3. The number of cleaved caspase-3-positive cells in the neuroepithelial layers was higher in PITRM1^−/−^ cerebral organoids than in the controls, starting at 2 months (Fig. 4f, g), suggesting a higher extent of cell death. No further increase in cell death was observed at later time points (Supplementary Fig. 4d). BTA-1 and thioflavin-T-positive aggregates were detected in cerebral organoids generated from PITRM1^−/−^ iPSCs, indicating that protein deposits are organized into amyloid-like aggregates, similar to those observed in AD plaques (Fig. 4h, i and Supplementary Fig. 4e, f). To analyze ubiquitin-dependent protein turnover, an immunostaining against ubiquitinated proteins was performed. PITRM1^−/−^ organoids display increased levels of ubiquitinated proteins (Fig. 4j, k). Next, we examined the expression levels of genes involved in UPR^mt^ in 2-month-old PITRM1^+/+^ and PITRM1^−/−^ cerebral organoids. PITRM1^−/−^ cerebral organoids exhibited a significant induction of UPR^mt^ transcripts (*ATF4, DDIT3, HSP60, HSPA9, ERO1*) (Fig. 4l). Moreover, gene expression of the mitochondrial proteases, *LONP1* and *CLPP*, was significantly upregulated in PITRM1^−/−^ cerebral organoids compared with controls (Fig. 4l).

### Inhibition of UPR^mt^ exacerbates Aβ proteotoxicity

**Fig. 5:**
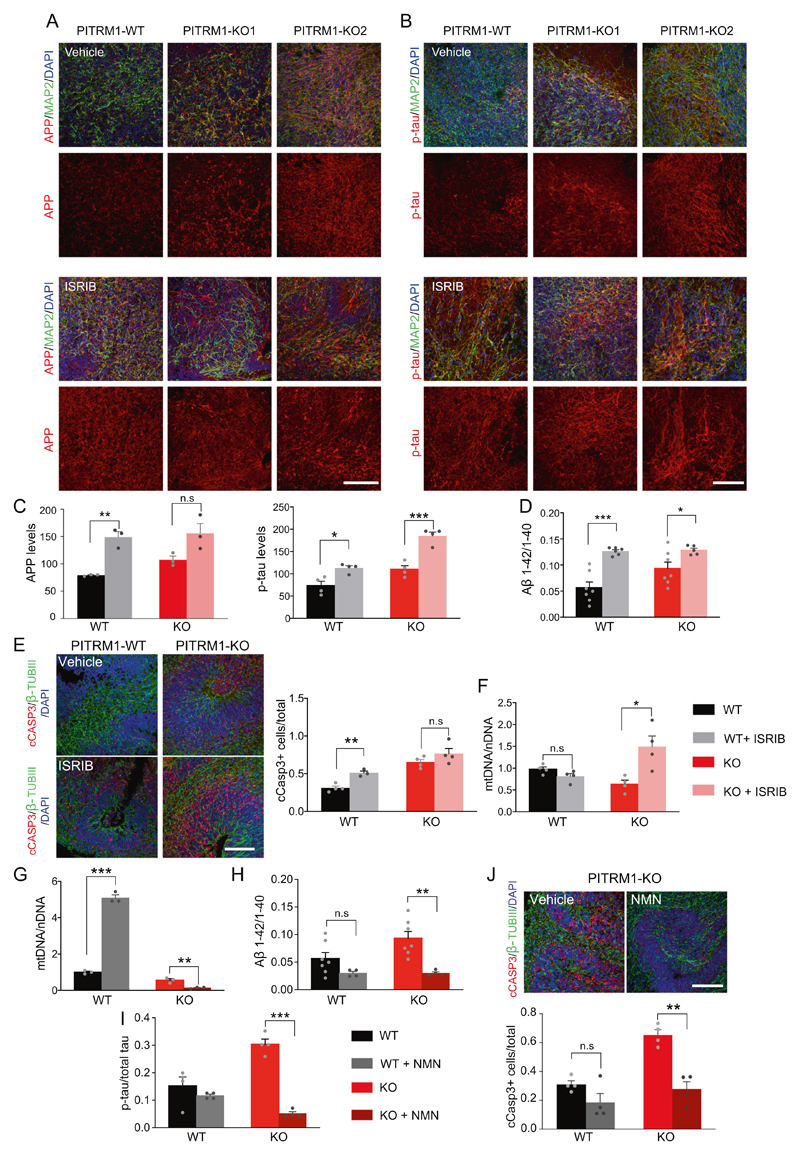
UPR^mt^ and mitophagy exert a protective role in PITRM1^−/−^ cerebral organoids. PITRM1^+/+^ and PITRM1^−/−^ cerebral organoids were treated with 500 nM ISRIB or vehicle, daily, from DIV 20 to DIV 50. **a**, **b** MAP2 (green), APP (red, in **a**), and phospho-tau (p-tau; red, in **b**) immunostaining in PITRM1^+/+^ and PITRM1^−/−^ cerebral organoids treated with ISRIB or vehicle. Representative confocal images are shown. Cell nuclei were counterstained with DAPI (blue). Scale bars, 100 μm. **c** Quantification of APP and phospho-tau mean fluorescent intensity (mean + SEM; *p < 0.05, **p < 0.01, ***p < 0.001, two-tailed *t* test, *n* = 3–4). **d** Quantification of Aβ species in the supernatant of PITRM1^+/+^ and PITRM1^−/−^ cerebral organoids treated with ISRIB or vehicle, as measured by Meso Scale immunoassay (mean + SEM; *p < 0.05, ***p < 0.001, two-tailed *t* test, *n* = 6–7). **e** Immunostaining for β-TUBIII (green) and cleaved caspase-3 (cCASP3, red) in PITRM1^+/+^ and PITRM1^−/−^ cerebral organoids treated with ISRIB or vehicle. Cell nuclei were counterstained with DAPI (blue). Scale bar, 100 μm. Quantification of the ratio of cCASP3-positive cells relative to the total number of β-TUBIII/DAPI cells is shown (mean + SEM; **p < 0.01, two-tailed *t* test, *n* = 3–4). **f** mtDNA content was measured in PITRM1^+/+^ and PITRM1^−/−^ cerebral organoids treated with ISRIB or vehicle as the mitochondrial (*16S*) to nuclear (*RPLP0*) DNA ratio by qRT-PCR (mean + SEM; *p < 0.05, two-tailed *t* test, *n* = 4). **g**–**j** PITRM1^+/+^ and PITRM1^−/−^ cerebral organoids were treated with 500 μM NMN or vehicle, daily, from DIV 45 to DIV 50. **g** mtDNA content was measured as the mitochondrial (*16S*) to nuclear (*RPLP0*) DNA ratio by qRT-PCR (mean + SEM; **p < 0.01, ***p < 0.0001, two-tailed *t* test, *n* = 3). **h** Aβ1–42/Aβ1–40 ratio in cerebral organoids measured by Meso Scale immunoassay (mean + SEM; **p < 0.01, two-tailed *t* test, *n* = 4–7). **i** ELISA assay measuring the levels of total and phospho-tau levels in cerebral organoid homogenates. The results are presented as the ratio of phospho-tau/total-tau (mean + SEM; ***p < 0.0001, two-tailed *t* test, *n* = 4). **j** Immunostaining and quantification for β-TUBIII (green) and cleaved caspase-3 (cCASP3, red) in PITRM1^−/−^ cerebral organoids treated with NMN or vehicle. Cell nuclei were counterstained with DAPI (blue). Scale bar, 100 μm. Analysis of the ratio of cCASP3-positive cells relative to the total number of β-TUBIII/DAPI cells is shown (mean + SEM; **p < 0.01, two-tailed *t* test, *n* = 4).

Given that UPR^mt^ can extend the lifespan in a variety of organisms [28–31], we asked whether the induction of UPR^mt^ observed in PITRM1^−/−^ cerebral organoids could act as a protective mechanism against defects of mitochondrial protein maturation and Aβ proteotoxicity. To this end, cerebral organoids were treated daily with ISRIB, a global ISR inhibitor [21, 32]. First, we examined APP levels and the phospho-tau/tau ratio by immunostaining. Consistent with the protective role of UPR^mt^ in our model, ISRIB-treated cerebral organoids showed higher APP and phospho-tau levels compared with controls (Fig. 5a–c). In parallel, ISRIB treatment significantly increased the Aβ42/Aβ40 ratio (Fig. 5d) in both PITRM1^+/+^ and PITRM1^−/−^ organoids. In line with these findings, ISRIB-treated PITRM1^+/+^ cerebral organoids show a significant increase in cleaved caspase-3-positive cells (Fig. 5e). Interestingly, ISRIB-treated cerebral organoids also showed an increase of mitochondrial DNA, suggesting that the inhibition of UPR^mt^ leads to a decrease in mitochondrial clearance (Fig. 5f).

### Enhancing mitophagy via NAD+ precursors ameliorates Aβ proteotoxicity

Because defects in mitophagy have been shown to contribute to AD [33], and ISRIB-treated cerebral organoids showed decreased mitochondrial clearance and the exacerbation of AD-like phenotypes, we investigated whether enhancing mitochondrial clearance ameliorates the AD-like phenotype in PITRM1-related mitochondrial neurodegeneration. To this end, PITRM1^+/+^ and PITRM1^−/−^ cerebral organoids were treated with the NAD+ precursor NMN, which has been shown to ameliorate mitochondrial function and clearance [34, 35]. While NMN supplementation resulted in an increased mtDNA/nDNA ratio in PITRM1^+/+^ organoids, a significant decrease in mitochondrial content was observed in PITRM1^−/−^ organoids after treatment (Fig. 5g). These data suggest a differential role of NAD+ boosters under physiological and pathological conditions—namely, the induction of mitochondrial biogenesis or the enhancement of mitochondrial clearance, respectively. Furthermore, NMN treatment significantly decreased the Aβ42/Aβ40 and phospho-tau/total tau ratios in PITRM1^−/−^ cerebral organoids as revealed by Meso Scale and ELISA measurements (Fig. 5h, i). Importantly, NMN treatment significantly decreased the number of cleaved caspase-3-positive cells in PITRM1^−/−^ cerebral organoids (Fig. 5j).

## Discussion

Because the brain is the organ with the highest energy demand, it comes as no surprise that it also represents the major disease target, both in genetically driven primary mitochondrial diseases as well as in common age-related neurodegenerative disorders. Despite this evidence, the causal link between mitochondrial demise and neurodegeneration remains elusive. We have recently reported that pathogenic variants in the nuclear-encoded mitochondrial peptidase PITRM1 result in childhood-onset recessive cerebellar disease characterized by spinocerebellar ataxia, mild intellectual disability, psychiatric manifestations, and cognitive decline [2, 3]. The clinical picture of these patients is unusual for mitochondrial disease, with a very slow progression of cognitive and psychiatric symptoms from childhood to their late sixties [2].

PITRM1^+/−^ mice show a neurological phenotype with the presence of Aβ-positive plaques in the neuropilum [2]. However, due to the embryonic lethality of complete PITRM1^−/−^ [2], the exact mechanisms of PITRM1 in brain function and disease could not be completely studied. To address these questions, we have generated a novel human stem-cell-based model of the loss of PITRM1 function that recapitulates several pathological aspects of human PITRM1-related mitochondrial and adult-onset neurodegenerative diseases. Employing iPSC-derived cortical neurons, we found that the loss of PITRM1 function leads to a strong induction of mitochondrial stress responses, enhanced autophagic flux and mitochondrial clearance, increased levels of APP and Aβ peptides, and an increased Aβ42/40 ratio. Several works have shown the uptake and accumulation of Aβ within the mitochondria in the postmortem brains of AD patients as well as in later stages of disease in APP transgenic mice [36, 37]. Furthermore, in vitro and yeast-based modeling has shown that the loss of PITRM1 function results in the incomplete degradation of Aβ in the mitochondria [2, 9]. Using sub-fractionation methods coupled with a highly sensitive immunoassay, we were unable to detect Aβ peptides in mitochondrial extracts from PITRM1-deficient cortical neurons. We cannot exclude the accumulation of a low amount of Aβ peptides within the mitochondria, below the detection limit of this study. We found that PITRM1^−/−^ neurons exhibit a significant decrease in mitochondrial membrane potential. However, PITRM1^−/−^ neurons did not show an increase in mitochondrial ROS production or defects in oxidative phosphorylation, which have been shown to be a direct effect of the accumulation of Aβ within mitochondria [38, 39]. Interestingly, the reduction of mitochondrial membrane potential was more evident in the neurites compared with the soma. Based on these results, we hypothesize that the mitochondrial pool in the neurites is the one which is most affected during the early stages of the diseases. Such localized changes may prevent the detection of significant changes in overall mitochondrial respiration.

Our data suggest that the imbalance of mitochondrial proteostasis can be the first event in the pathogenetic cascade in PITRM1-related neurological syndrome. APP accumulation observed in PITRM1^−/−^ neurons and cerebral organoids may be a consequence of the overload of the proteasome in response to mitochondrial protein misfolding. The ubiquitin–proteasome system (UPS) is also involved in the quality control of mitochondrial proteins, especially mitochondrial precursor proteins and proteins of the outer membrane [40]. Supporting an overload of the UPS system, we detected an accumulation of ubiquitinated proteins in PITRM1-deficient neurons and cerebral organoids. Based on these data, we propose that mitochondrial proteotoxic stress, possibly linked to the accumulation of non-degraded MTS as a result of PITRM1 dysfunction and the accumulation of unprocessed mitochondrial proteins, triggers a cytosolic response with overload and saturation of the proteasome and defects in cytosolic protein degradation.

PITRM1 deficiency led to a strong induction of UPR^mt^ in both 2D and brain-organoid model systems. UPR^mt^ is a transcriptional response involving mitochondrial chaperones and proteases activated by mitochondrial dysfunction and defects in mitochondrial protein folding [41]. It is a key cellular quality control mechanism that promotes the maintenance of mitochondrial health and ensures proper cellular functions [42]. Despite the evidence of UPR^mt^ activation in the aging and diseased brain [20], whether and how UPR^mt^ contributes to neurodegenerative processes is unclear. UPR^mt^ has been proposed as being a double-edged sword, with its chronic activation leading to detrimental consequences for cellular and organismal function [22, 43]. A detrimental role of UPR^mt^ has been demonstrated in animal models of mitochondrial diseases [22, 43]. However, mitochondrial stress responses have been documented in AD and recent work has shown that enhancing UPR^mt^ provides protections against Aβ proteotoxicity [21, 44]. In line with a beneficial role of UPR^mt^, PITRM1^−/−^ cerebral organoids treated with ISRIB, an inhibitor of the ISR, showed a significant increase of APP levels, an increased Aβ42/Aβ40 ratio, tau hyperphosphorylation and cell death. Based on these data, we propose that PITRM1-related induction of UPR^mt^ is a protective mechanism against proteotoxic stress at both the mitochondrial and cytosolic levels. Importantly, our findings indicate that the consequences of chronic mtISR upregulation may vary substantially among different mitochondrial diseases and that the underlying molecular defect should be carefully considered for therapeutic decisions.

Though UPR^mt^ was activated in both 2D and 3D PITRM1 KO models, only PITRM1 KO cerebral organoids displayed the typical abnormalities observed in the brains of AD patients, including neuronal cell death, tau pathology, and the accumulation of protein aggregates, similar to Aβ plaques. On the other hand, despite APP accumulation and an increased Aβ42/Aβ40 ratio, we did not detect overt cell death, nor did we detect Aβ aggregates or tau pathology in 2D iPSC-derived neuronal cultures. These findings indicate that 3D systems, as compared with 2D systems, provide a more relevant disease model that is advantageous in investigating the link between cellular proteostasis and disease. Due to the prolonged culturing conditions, as well as the presence and interaction among different cell types, including glial cells, 3D model systems may promote the development of disease-relevant phenotypes such as protein aggregation and neuronal death [45]. Combining cerebral organoids with scRNA-seq, we were able to demonstrate the cell-type-specific impact of the loss of PITRM1 function. PITRM1 has a strong impact on pathways related to mitochondrial function (oxidative phosphorylation, mitochondrial dysfunction, sirtuin signaling, and the NRF2-mediated oxidative stress response). Interestingly, this effect was observed in all the major brain cell types except for progenitor cells. In line with a role for immune pathways in AD pathology, astrocyte signatures also markedly differed between PITRM1^+/+^ and PITRM1^−/−^ cerebral organoids with significant dysregulation of immune-related pathways. These data highlight mitochondrial dysfunction as an important driver of immune reactions. Though microglia emerged in our scRNA sequencing analysis and previous reports show the development of microglia within cerebral organoids [46], the low number of cells identified may have hampered the identification of microglial relevant pathways in our primary mitochondrial disease organoid model.

With respect to the mechanisms, our data suggest that PITRM1 deficiency triggers compensatory quality control mechanisms at both the cytosolic and mitochondrial levels (i.e., the induction of UPR^mt^ and autophagy/mitophagy) that ensure the maintenance of cellular proteostasis. However, over time, these mechanisms may not be sufficient to protect neuronal cells against mitochondrial proteotoxicity or may overload cytosolic quality control pathways, such as the UPS, as observed in long-term culture cerebral organoids.

Several findings—including the induction of autophagic flux, decreased mtDNA levels, and an increase of mitochondrial protein ubiquitination—suggest that PITRM1 deficiency leads to increased mitochondrial clearance. It is known that defects in PITRM1 activity lead to impaired MTS processing and the accumulation of MTS and precursor proteins that have a toxic effect on mitochondria. In line with this evidence, we report that PITRM1^−/−^ neurons show defects in the maturation of the human frataxin precursor and decreased mitochondrial membrane potential. Enhanced mitophagy could be triggered by mitochondrial depolarization in response to MTS accumulation within mitochondria. Furthermore, UPR^mt^—and, in general, the ISR—activates the autophagic pathway [24, 25]. Interestingly, inhibition of ISR resulted in an increase in mtDNA, suggesting that the activation of UPR^mt^ is linked to the enhanced mitochondrial clearance in our model.

Mitochondrial stress response and mitophagy transcripts have been found to be upregulated in mild cognitive impairment as well as in mild and moderate AD patients, whereas defective mitophagy may play a role in the disease progression at later stages [33, 47]. Fang et al. have recently shown that the enhancement of mitophagy is able to rescue AD-related pathology in different AD model systems [33]. In line with this finding, we show that stimulating mitophagy with NMN, an NAD+ booster, significantly improves mitochondrial clearance, with a reduction of the Aβ42/Aβ40 ratio, tau hyperphosphorylation and neuronal loss. On the other hand, inhibition of UPR^mt^ with ISRIB led to decreased mitochondrial clearance and the aggravation of Aβ and tau pathology. Thus, our data suggest that mitophagy has a protective role against mitochondrial proteotoxicity induced by PITRM1 deficiency. Interestingly, NMN-related induction of mitophagy was evident only in PITRM1^−/−^ cerebral organoids, while induction of mitochondrial biogenesis was detected in PITRM1^+/+^organoids upon treatment.

In conclusion, we report a novel cellular model of human PITRM1 deficiency that recapitulates several fundamental pathological aspects of PITRM1-related mitochondrial disease. Using human iPSC-derived cortical neurons and cerebral organoids, we show that the loss of PITRM1 function leads to pathological features similar to those observed in AD—namely, protein aggregation, tau hyperphosphorylation, and neuronal death. We report that PITRM1 deficiency induces the impairment of mitochondrial proteostasis and the activation of UPR^mt^, which activates cytosolic quality control pathways such as the UPS and autophagy. The overload of the UPS causes, in the long run, a reduced capacity of degrading cytosolic proteins leading to APP accumulation, an increased level of Aβ species, an increased Aβ42/40 ratio, and extracellular protein aggregation. Furthermore, we show that, similar to what has been described in AD, enhancing UPR^mt^ and mitophagy ameliorates neuropathological features in primary mitochondrial disease-related neurodegeneration. Importantly, although human PITRM1 mutations are relatively rare, the disease mechanisms described in the present study may apply to both primary mitochondrial diseases and more common adult-onset neurological diseases. Thus, our data support a mechanistic link between primary mitochondrial disorders and common neurodegenerative proteinopathies.

## Supplementary Material

The online version of this article (https://doi.org/10.1038/s41380-020-0807-4) contains supplementary material, which is available to authorized users.

Supplementary figures

Supplementary table 1

Supplementary table 2
