# Supplementary material for: Loss of function of the mitochondrial peptidase PITRM1 induces proteotoxic stress and Alzheimer’s disease-like pathology in human cerebral organoids
Source: Mol Psychiatry. Author manuscript; Available in PMC 2022 Jan 17. (PMC8758476; doi:10.1038/s41380-020-0807-4)
Supplement: Supplementary figures [file EMS135869-supplement-Supplementary_figures.pdf]

Supplementary figure legends.

Supplementary Figure 1. Generation of PITRM1 knockout human iPSC lines by CRISPR-Cas9. (A) Schematic overview of CRISPR/Cas9-mediated *PITRM1* knockout in human iPSCs. Two single-guide RNAs (sgRNAs) targeting exon 3 and exon 4 of *PITRM1* were used, leading to a deletion that resulted in an early stop codon. **(B)** Analysis of *PITRM1* exon deletion on genomic DNA by PCR gel electrophoresis. **(C)** *PITRM1* mRNA expression in WT and PITRM1$^{-/-}$ (KO) iPSCs (mean +SEM; * p<0.05, two-tailed t-test, n=3). **(D, E)** Representative Western blot for PITRM1, showing the absence of PITRM1 in PITRM1$^{-/-}$ iPSCs, neural precursor cells (NPCs), and iPSC-derived cortical neurons (clones #1, #2). **(F, G)** Isogenic PITRM1$^{+/+}$ and PITRM1$^{-/-}$ iPSC-derived neurons were loaded with the Fluo-4 AM fluorescent dye to determine changes in cytosolic calcium and treated with 0.6 mM KCl. Representative images are shown. Scale bar, 10 μm. (G) Average traces of Fluo4 AM fluorescence changes in isogenic PITRM1$^{+/+}$ and PITRM1$^{-/-}$ iPSC-derived neurons stimulated with 0.6 mM KCl (mean + SEM, n=3). **(H)** LDH release was assessed in PITRM1$^{+/+}$ and PITRM1$^{-/-}$ iPSC-derived neurons at DIV 35 and DIV 65. LDH release values were calculated as the percentage of untreated cells lysed by incubation with Triton X-100 (mean +SEM; n=3). **(I, J)** Western blot analysis of frataxin protein processing in NPCs and iPSC cortical neurons. The quantification of mature (mF) and intermediate (iF) forms of frataxin in iPSC-derived cortical neurons is shown in (J) (mean +SEM; * p<0.05, two-tailed t-test, n=4).

Supplementary Figure 2. Characterization of the mitochondrial and autophagic function in PITRM1 iPSC-derived cortical neurons. (A) Mitochondrial reactive oxidative species level as analyzed by MitoSOX labeling in isogenic PITRM1$^{+/+}$ and PITRM1$^{-/-}$ iPSC-derived neurons (mean + SEM, n=7). **(B)** Oxygen consumption rate (OCR) of PITRM1$^{+/+}$ and PITRM1$^{-/-}$ iPSC-derived neurons. Data are normalized to protein content (mean ± SEM, n=6). **(C, D)** Western blot analysis of OXPHOS complex protein levels in PITRM1$^{+/+}$ and PITRM1$^{-/-}$ iPSC-derived neurons. Representative blot is shown in (C) and the quantification is shown in (D) (mean + SEM; * p<0.05, two-tailed t-test, n=3-7). **(E)** Immunostaining of PITRM1$^{+/+}$ and PITRM1$^{-/-}$ iPSC-derived cortical neurons for LC3 (green) and β-TUBIII (red). Nuclei were counterstained with DAPI (blue). Scale bars, 10 μm. **(F)** Number of LC3-positive vesicles per β-TUBIII-positive cell relative to the control neurons (mean + SEM; *** p<0.001, two-tailed t-test, n=3). **(G)** NeuO (green) and BTA-1 (blue)

staining in PITRM1$^{+/+}$ and PITRM1$^{-/-}$ iPSC-derived neurons at DIV 50. Representative confocal images are shown. Scale bars, 10 μm. **(H)** Representative Western blots of phospho-tau (Thr231, Ser202 and Thr205, Thr181 phosphorylation sites) and total tau in PITRM1$^{+/+}$ and PITRM1$^{-/-}$ neurons at DIV 50; total tau and β-Actin were used as the loading controls. **(I)** Quantification of phospho-tau protein levels relative to the loading control total tau/β-Actin (mean + SEM; n=3).

Supplementary Figure 3. scRNA-seq of cerebral organoids reveals a cell-type specific impact of PITRM1. (A) Representative bright field images of PITRM1$^{+/+}$ and PITRM1$^{-/-}$ cerebral organoids at DIV 9 and DIV 25. Scale bars, 200 μm. **(B, C)** UMAP projection of all organoid cells from PITRM1$^{+/+}$ and PITRM1$^{-/-}$ cerebral organoids. In (B), the UMAP projection is colored by cell cycle stages in the different clusters. (C) shows the expression of classical cell type-specific markers: *STMN2* (neurons), *SLC1A3* (radial glia), *TOP2A* (NPC), *CEBPB* (glia), *S100B* (astrocytes), and *TMEM119* (microglia). Gene expression data were normalized and transformed using a natural logarithm. **(D)** TNF-α and IL-1β levels in cerebral organoids, as assessed by ELISA. The protein concentration was measured by BCA and equal amounts of total protein were used (mean + SEM; **** $p<0.0001$, two-tailed t-test, n=5).

Supplementary Figure 4. PITRM1$^{-/-}$ cerebral organoids show AD-like pathology. **(A)** MAP2 (green), APP (red, left panel), and phospho-tau (red, right panel) immunostaining in cerebral organoids. Representative confocal images are shown. Cell nuclei were counterstained with DAPI (blue). Scale bars, 100 μm. **(B)** Quantification of APP and phospho-tau fluorescent intensity in 2-month old cerebral organoids (mean + SEM; *$p<0.05$, two-tailed t test, n=3-4). **(C)** MAP2 (green) and phospho-tau (red) immunostaining in PITRM1$^{+/+}$ and PITRM1$^{-/-}$ cerebral organoids at 60, 75, and 180 days. Representative confocal images are shown. Cell nuclei were counterstained with DAPI (blue). Scale bars,100 μm. (**D**) Quantification of cCASP3 positive cells relative to the total number cells, measured by DAPI staining in 1-, 2-, and 6-month old cerebral organoids (mean + SEM; * $p<0.05$, ***$p<0.001$, two-tailed t test, n=3). **(E)** Thioflavin T (ThioT) (green) immunostaining in 2-month old PITRM1$^{+/+}$ and PITRM1$^{-/-}$ cerebral organoids. Representative confocal images are shown. Cell nuclei were counterstained with DAPI (blue). Scale bars, 100 μm. **(F)** Quantification of ThioT fluorescent intensity in 2-month old cerebral organoids (mean + SEM; * $p<0.05$, two-tailed t test, n=3).

Table S1. Cell cluster analysis: differentially expressed genes. Statistics by Wilcoxon-rank-sum test (p-value adjustment by Bonferroni correction).

Table S2. Differentially expressed genes by cell cluster in PITRM1$^{+/+}$ and PITRM1$^{-/-}$ cerebral organoids. Statistics by Wilcoxon rank-sum test (p-value adjustment by Bonferroni correction).

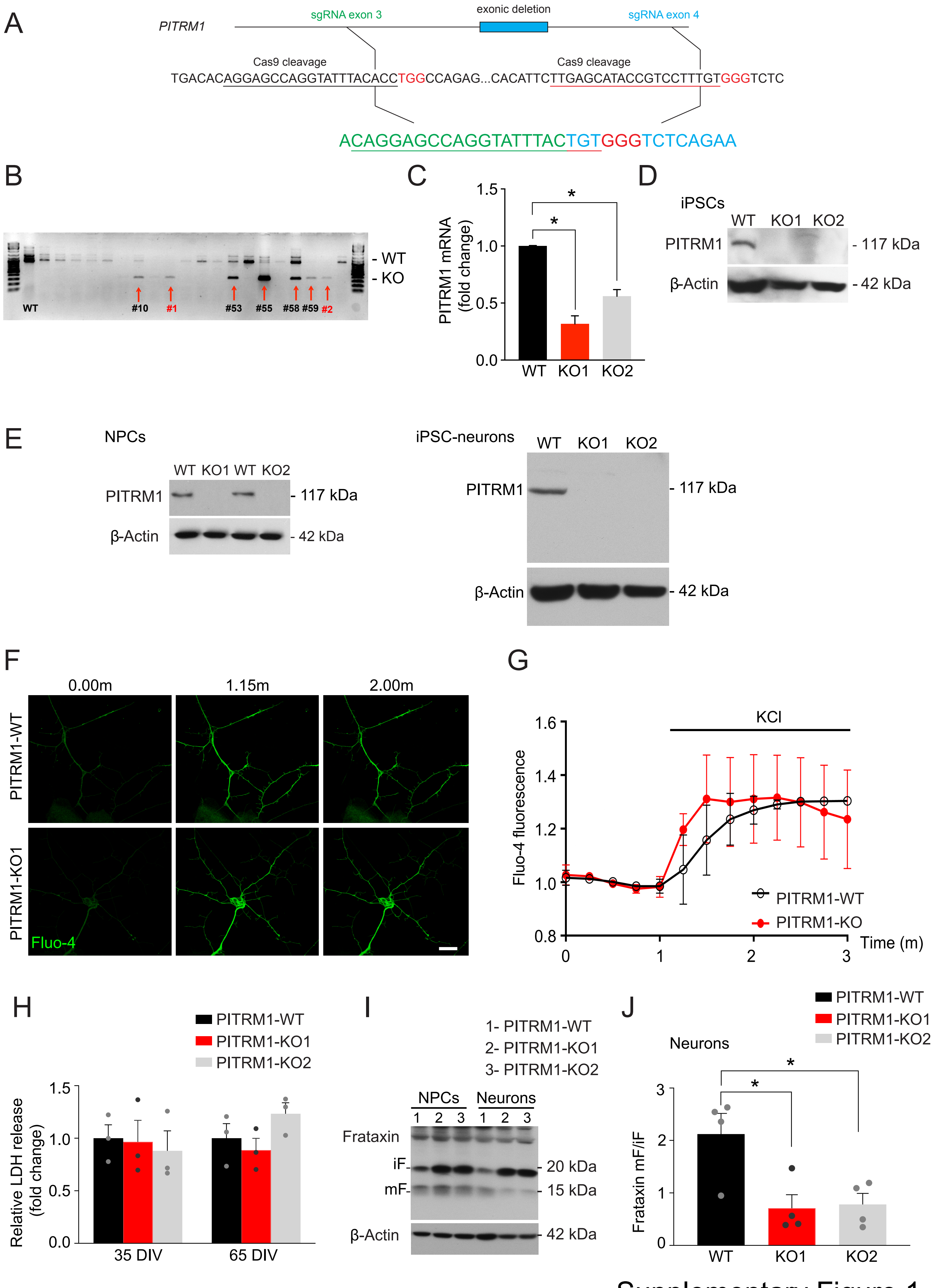

Supplementary Figure 1

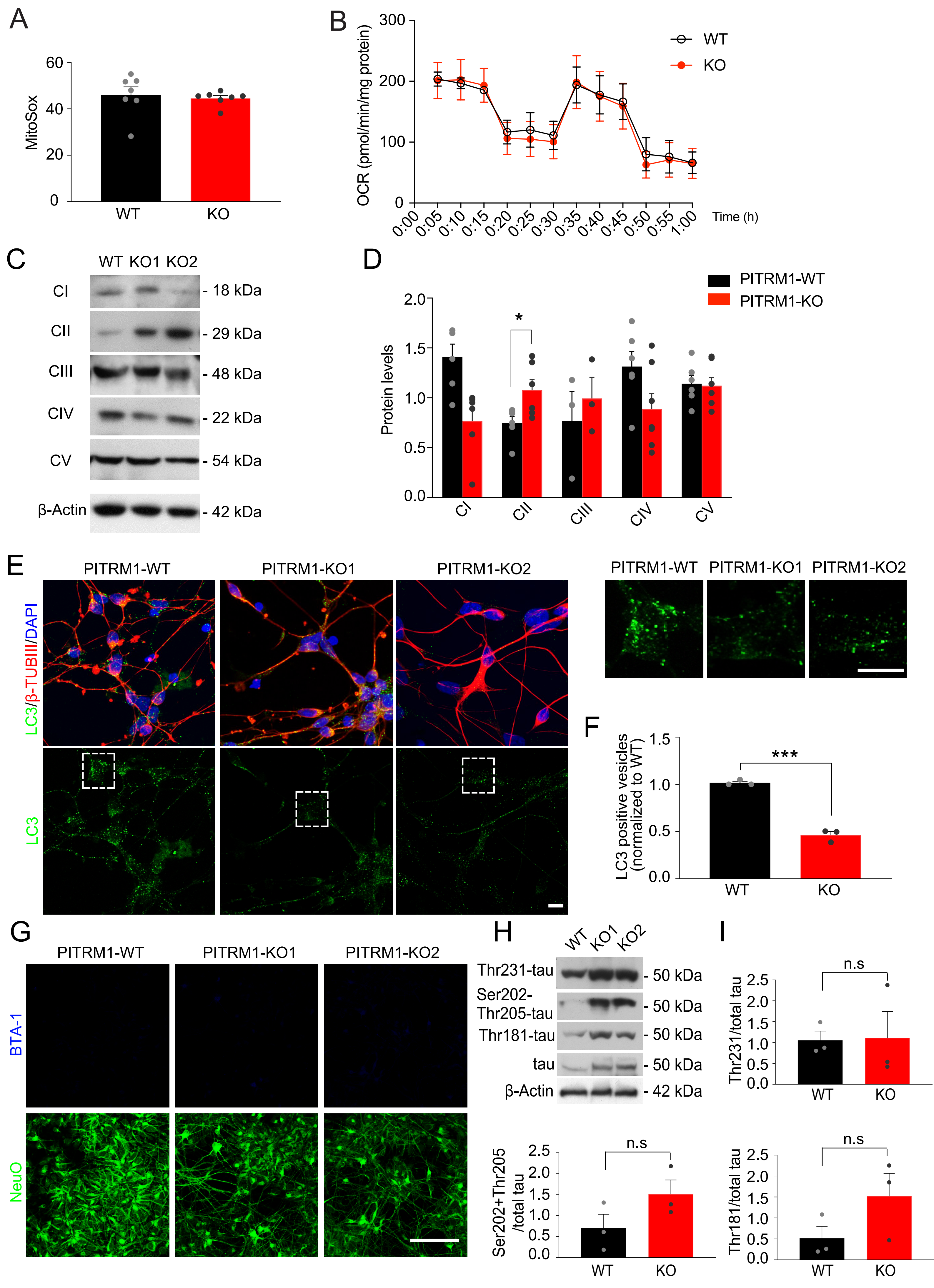

Supplementary Figure 2

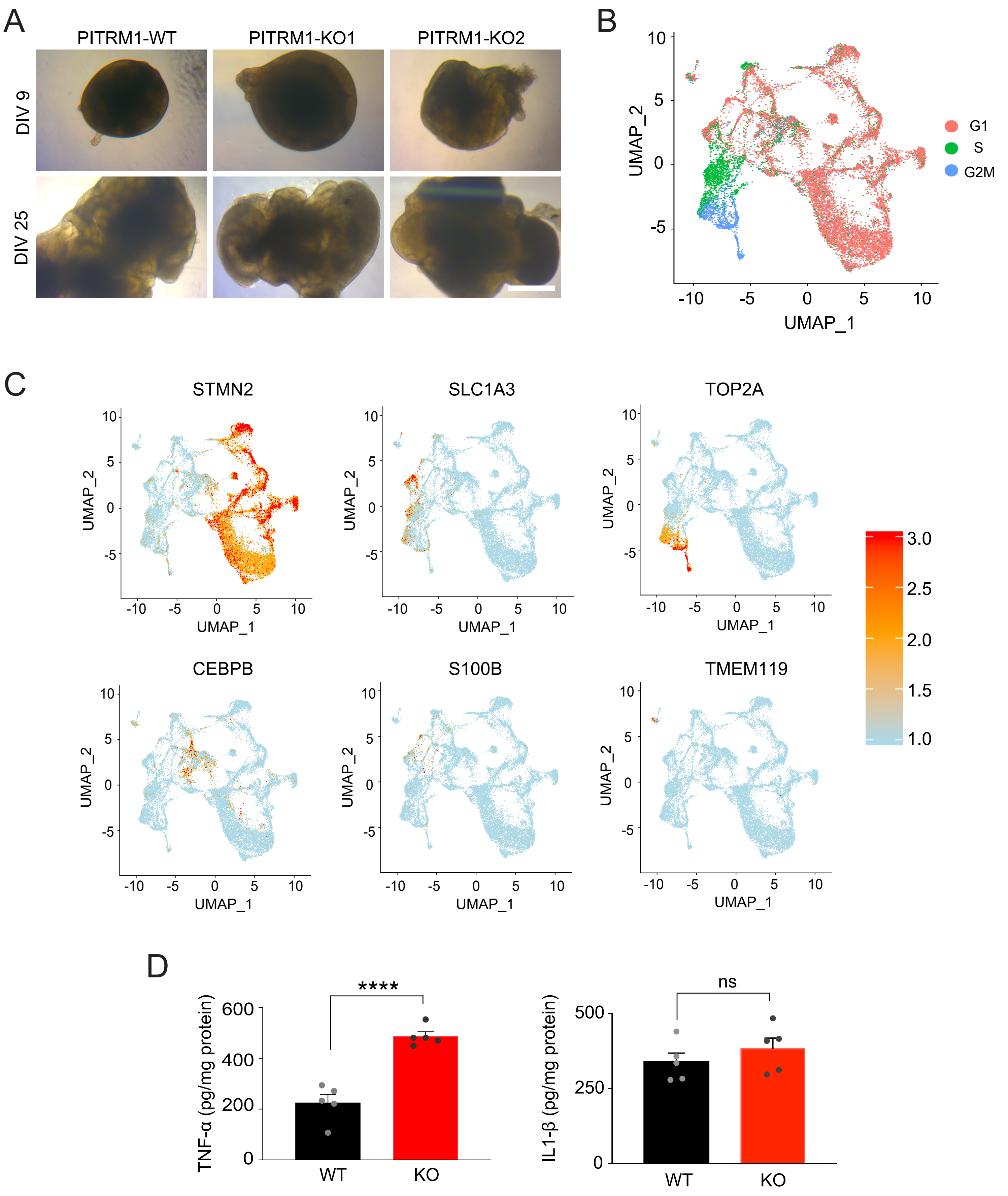

Supplementary Figure 3

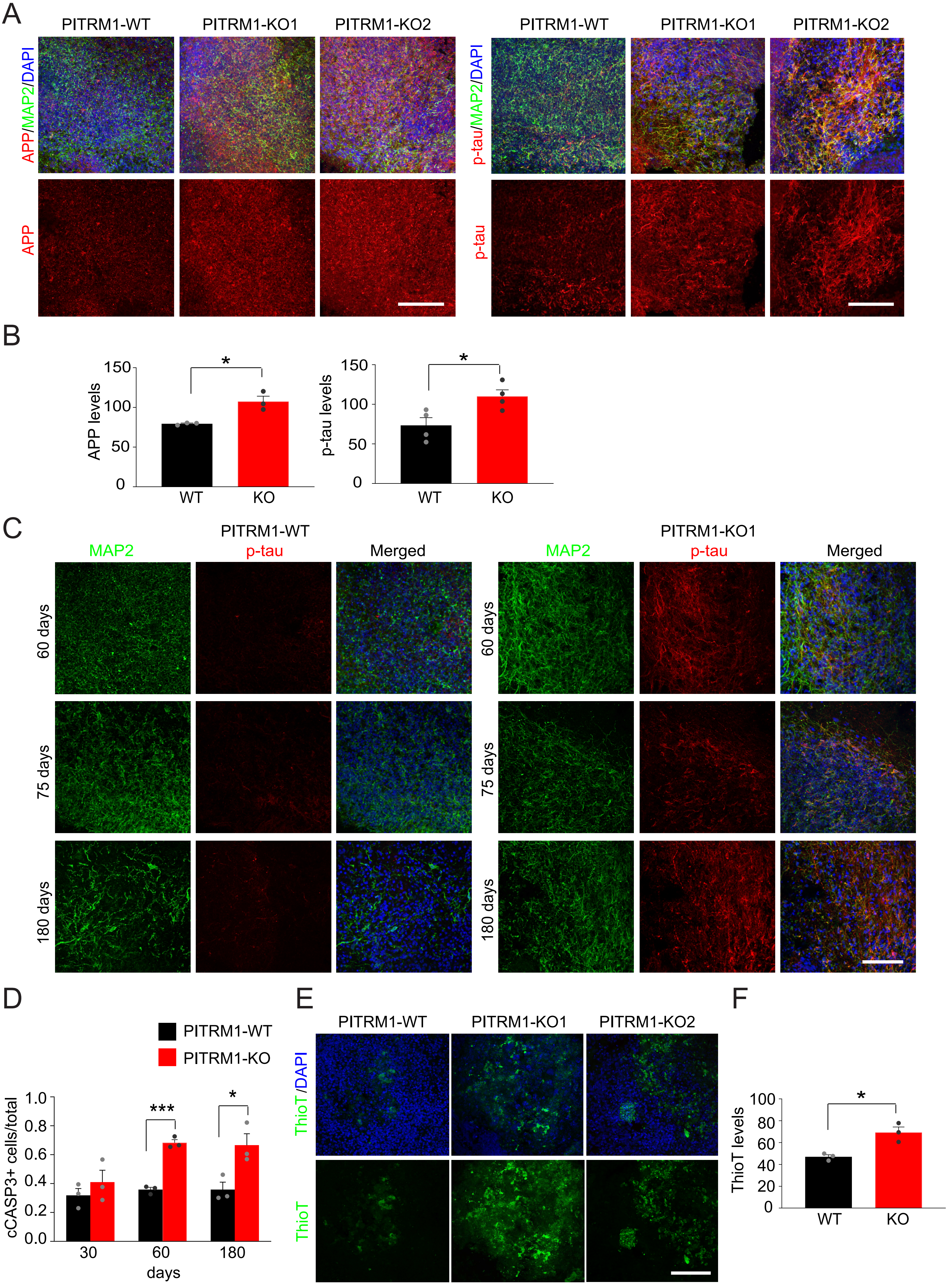

Supplementary Figure 4
